# The Potential Impact of White-Nose Syndrome on the Conservation Status of North American Bats

**DOI:** 10.1371/journal.pone.0107395

**Published:** 2014-09-09

**Authors:** Davi M. C. C. Alves, Levi C. Terribile, Daniel Brito

**Affiliations:** 1 Laboratório de Ecologia Teórica e Síntese, Departamento de Ecologia, Universidade Federal de Goiás, Goiânia, Goiás, Brasil; 2 Laboratório de Macroecologia, Universidade Federal de Goiás, Jataí, Goiás, Brasil; 3 Laboratório de Ecologia Aplicada e Conservação, Departamento de Ecologia, Universidade Federal de Goiás, Goiânia, Goiás, Brasil; University of Regina, Canada

## Abstract

White-Nose syndrome (WNS) is an emergent infectious disease that has already killed around six million bats in North America and has spread over two thousand kilometers from its epicenter. However, only a few studies on the possible impacts of the fungus on bat hosts were conducted, particularly concerning its implications for bat conservation. We predicted the consequences of WNS spread by generating a map with potential areas for its occurrence based on environmental conditions in sites where the disease already occurs, and overlaid it with the geographic distribution of all hibernating bats in North America. We assumed that all intersection localities would negatively affect local bat populations and reassessed their conservation status based on their potential population decline. Our results suggest that WNS will not spread widely throughout North America, being mostly restricted to the east and southeast regions. In contrast, our most pessimistic scenario of population decline indicated that the disease would threaten 32% of the bat species. Our results could help further conservation plans to preserve bat diversity in North America.

## Introduction

Diseases are one of the major causes of biodiversity homogenization [1], [2]. Several vertebrate species have been considered threatened due to the impacts of diseases dynamics. For instance, Chytridiomycosis is a disease caused by the invasive fungus *Batrachochytrium dendrobatidis*, being a major cause of amphibian population decline and extinction worldwide [3], [4]. Several species of New World birds have been suffering severe die-offs from the invasion of the West Nile Virus [5], [6]. Deaths of millions of individuals, over 50% population declines, and local extinctions of several populations have been reported [7].

Until recently, diseases were not a major threat to mammals [8], since only a few mammal species were particularly threatened by emerging infectious diseases [9]. However, this picture is changing as a new disease spreads among North American bats. White-Nose Syndrome (WNS) affects hibernating bats in North America and was first detected in a cave in the state of New York, in the 2006–2007 winter [10]. WNS is caused by the European fungus *Pseudogymnoascus destructans*; it colonizes the hairless skin of hosts and grows better in low ambient temperatures [11], [12]. The mortality mechanism is associated with the increase in bat arousals to normothermia from torpor during hibernation, which prematurely expends the energy necessary to maintain bats during winter [13].

The WNS has already killed roughly six million bats having spread over two thousand kilometers within North America [14], [15]. The species suspected or already infected by the pathogen are: *Eptesicus fuscus, Myotis leibii, M. lucifugus, M. septentrionalis, M. sodalis, Pipistrellus subflavus,* and *M. grisescens* [16]. Unfortunately, *M. sodalis* and *M. grisescens* have experienced drastic population declines before the onset of WNS, and while the International Union for the Conservation of Nature and Natural Resources (IUCN) have not evaluated them against WNS, they are already classified as Vulnerable and Near Threatened, respectively [17]. The United States Fish and Wildlife Service (USFWS) produced a national plan to manage the disease comprising research and management actions on epidemiology and wildlife management [18]. Regardless of important advances in understanding the epidemiology of WNS [13] and the records of severe population declines due to WNS [19], [20], [21], [22], little is known about impacts of the disease on the conservation status of such bat species.

A critical issue to predict the spread of an invasive disease such as WNS is to understand the dynamics of its spatial distribution, which reflects large-scale processes, such as environmental factors acting on its dispersion [23]. For instance, where the pathogen has successfully established within its native or current range could be used to identify locations that has not been invaded yet but might be at risk [24]. Consequently, host populations that inhabit such locations might be affected by the disease invasion. In this sense, a particularly useful approach is the Ecological Niche Modeling (hereafter ENM; also called potential distribution modeling), which encompasses a set of tools relating known occurrences of species or phenomena (in this case, the disease) to geographic information system layers that summarize variation in several environmental dimensions [25]. The result from this relationship could be extrapolated to characterize the potential geographic distribution of invasive pathogens benefiting the identification of areas where native host species might become subjected to the disease [25].

Thus, our main objective was to assess whether predicting the spread of *P. destructans* in North America could represent an impact strong enough to negatively affect the current conservation status of North American hibernating bats. We believe this information will help conservationists and decision makers allocate their efforts to avoid the disease spread.

## Methods

### Modeling the invasion of WNS in the North America

We used MaxEnt (version 3.3.3e) as our ENM to establish the potential geographic spread of WNS throughout North America. MaxEnt is a machine-learning method based on a principle from statistical mechanics and information theory [26]. It states that the most spread out and close to uniform probability distribution subject to known constraints is the most satisfactory approximation for an unknown distribution; the resulting prediction is interpreted as the relative likelihood of habitat use, or environmental suitability [26]. This method has generated great interest for having higher predictive accuracy than other methods when applied to “presence-only” occurrence data [27], as is the case in our study.

Since our main objective using ENM was to predict the potential spread of WNS in the invaded area (North America), we follow the approach proposed by Peterson and Soberón [28]. We establish the native area for the pathogen (Europe; [11]) as the region where the fungus presented the historical ability to disperse and select available habitats [28]. We used the points from this area to calibrate the model, numbering 51 sites for the occurrence of *P. destructans,* whereas 18 presences were confirmed with genetic analyses, and 33 were suspected both by photographic or visual records ([29], Table S1). We decided to include the occurrences suspected for *P. destructans* because a fungus with a longstanding interaction with their host is very conspicuous and relative easy to identify, which is the case in Europe, in which both pathogen and host are assumed to have coevolved. We establish the invaded area for the disease (North America; [11]) as the region the pathogen was able to colonize only recently. To validate the model, we used the occurrence points of this area, with a total of 184 confirmed sites for the occurrence of *P. destructans* ([30]; SupInf1; updated until March 2014), and 1,000 randomly selected background points of the invaded region. The benefit of this validation approach is the use of two independent groups of occurrence records, one group for calibration (European points) and the other for validation (North American points). We used the area under the curve (AUC) of the receiver operating characteristics (ROC) as validation metric. The model projection was conducted just upon the invaded area, characterizing the potential distribution for WNS. In other words, the region with adequate abiotic conditions for the disease to occur, but without the necessary time to be fully colonized by the pathogen [28]. We assumed that biotic interactions that cause density-dependent effects are not important to establish WNS geographic distribution at the spatial scale of our study [28].

The *P. destructans* niche was constructed using six environmental variables: (1) annual temperature range; (2) mean temperature of the wettest quarter; (3) precipitation of the wettest month; (4) precipitation of the driest month; (5) elevation standard deviation; and (6) land classes (variables 1–4 obtained from [31] and [32]; variable five obtained from [33]; and variable six obtained from [34]). The rationale for choosing these variables followed [35], which quantified the environmental factors that most satisfactorily explained the actual WNS geographic distribution. The resolution of all variables was 0.16 degrees. Since part of these variables is spatially auto-correlated affecting the ENM results [36], we used environmental filters to select the occurrence points with greater environmental distance in the native region [37]. We used the annual temperature range and altitude as filters with a resolution of 10 and 5 units, respectively, decreasing native occurrences to 43 environmentally equidistant points.

We transformed the potential distribution map for WNS including the continuous variable environmental suitability into a categorical distribution map (CPD). We used the maximum test sensitivity. plus the specificity threshold to generate CPD featuring the smallest costs with omission and commission errors.

### Assessing the impact of WNS spread on bat conservation status

We used the abovementioned CPD of *P. destructans* to estimate the potential impact of WNS upon the North American susceptible hosts. All North American hibernating bats (25 species) were considered susceptible hosts according to [16] (see the map of the susceptible host’s richness in SupInf 2). Thus, we transformed the occurrence extension (EOO) of the 25 bat species (obtained from [17]) into 0.5° resolution polygons. We overlapped the CPD with the EOO map of each bat species, and then subtracted the former by the latter (see Figure S1 for an explanation).

We assumed three scenarios of population decline: Pessimistic, Intermediate, and Optimistic. All of them were based on mortality rates obtained by field surveys within caves for *Myotis lucifugus* (0.3–0.99; mean = 0.73; [19]). The Intermediate scenario assumes that the intersection of a grid cell in which a given species occurs with a grid cell in which *P. destructans* occurs, reduced the local population of a given host in 73%. For instance, we assumed that the local population of species A in cell Z = 1. The disease effect upon species A on that cell is 0.73 and then the remaining population of species A on cell Z is 0.27 of the original population. We then summed the values of all intersecting cells and divided it by the amount of cells in which species A occurred. In the Pessimistic scenario, we used a population decline parameter of 99%, and in the Optimistic scenario, a population decline scenario of 30%.

We used these parameter values of population declines because all potential or actual hosts for WNS come from the same family of *M. lucifugus* (Vespertilionidae). Moreover, the genus *Myotis* corresponds to 64% of all potential or actual hosts for WNS. The parameter values (30–99%) are in accordance with a study conducted on northeastern populations for several *Myotis* species [20], also boarding a recently estimation of population decline of 80% for all actual hosts for WNS in North America ([18]; accessed in 04-2014).

Extinction risk assessments, such as the one supported by the IUCN, is a transparent and objective methods to predict the possibility of a species to disappear. They are generally based on species’ geographic range and\or population decline due to threat factors [38]. Thus, to quantify the impact of WNS on the conservation status of a given bat species, we used all three potential population decline scenarios caused by the disease to assess the species against criterion A (criterion A4ce) of the Red List of IUCN [38]. More specifically, criterion A4ce represents an estimated population decline, whose causes might not have ceased, being based on a decline in occurrence extent due to pathogens effects. According to this criterion, if the estimated population decline is equal or higher than 30%, the species is classified as Vulnerable (VU); equal or higher than 50% as Endangered (EN); equal or higher than 80% as Critically Endangered (CE; [38]).

All analyses were carried out using R (www.r-project.org). ENM analyses were conducted using dismo package [39] and shapefiles and rasters manipulations were performed in letsR package [40].

## Results

**Figure 1:**
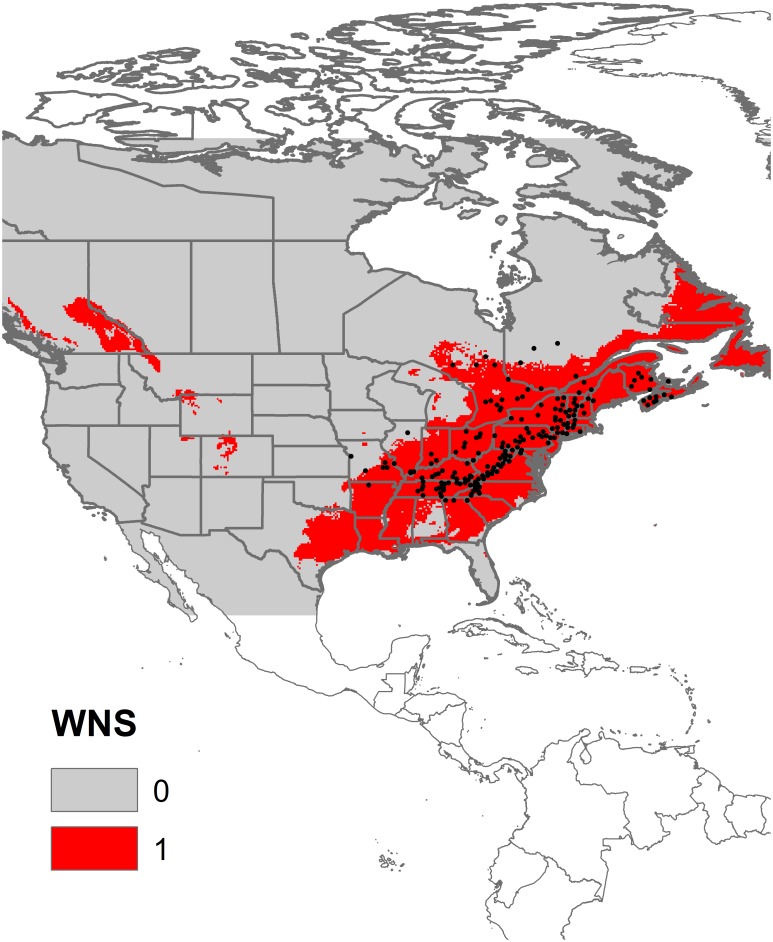
The potential spread of White-Nose Syndrome in North America. The black dots are the current occurrence data of the pathogen *P. destructans*.

Our map of the potential spread of WNS in North America reflected the current distribution of the disease, concentrated in the Appalachian Mountains, but indicated potential areas susceptible to invasion towards coastal areas of northeastern North American, the Mississippi basin, and the Rocky Mountains (Fig. 1; AUC 0.94). Land classes (76%) and precipitation of the driest month (21%) represented the most contributing variables to the model; the remaining variables presented very small contributions (less than 1% each).

The Pessimistic scenario of population decline revealed that 36% (nine species) of North American bat species susceptible to WNS are expected to lose over 25% of their entire population (*C. rafinesquii*, *E. fuscus, M. austroriparius, M. grisescens*, *M. leibii*, *M. septentrionalis*, *M. sodalis, Nycticeius humeralis,* and *P. subflavus*; Table 1). Among these species, eight bat species would suffer severe rates of population decline enough to be classified as one of the IUCN’s threat categories; five of these species would present rates of population decline higher than 80% (Critically Endangered species; Table 2). The Intermediate scenario revealed that 32% (eight species) would be classified as one of the IUCN’s threat categories, and the Optimistic scenario revealed that only *M. sodalis* would be classified in a threat category (Table 2).

**Table 1:** The expected relative population reduction of the North American bats susceptible to the White-Nose syndrome spread according to three population-reduction scenarios.

		Scenarios	
Species	Pessimistic	Intermediate	Optimistic
*Myotis leibii*	96.6	71.2	29.3
*Myotis grisescens*	83	61.1	25.1
*Corynorhinus rafinesquii*	80	59	24.2
*Myotis austroriparius*	77.4	57.1	22.4
*Myotis sodalis*	76.3	56.3	23.1
*Nycticeius humeralis*	61.8	45.5	18.7
*Pipistrellus subflavus*	61.6	45.4	18.6
*Myotis septentrionalis*	42.4	31.3	12.9
*Eptesicus fuscus*	25.2	18.5	7.6
*Myotis lucifugus*	22.9	16.9	6.9
*Myotis vellifer*	9.3	6.8	2.8
*Corynorhinus townsendii*	6.9	5.1	2.1
*Myotis evotis*	6.9	5.1	2.1
*Euderma maculatum*	5.9	4.3	1.8
*Myotis volans*	5.1	3.7	1.5
*Myotis californicus*	4.2	3.1	1.3
*Myotis yumanensis*	4.1	3	1.2
*Myotis ciliolabrum*	3.6	2.7	1.1
*Myotis keenii*	3.6	2.6	1.1
*Antrozous pallidus*	2	1.3	0.5
*Myotis thysanodes*	0.8	0.6	0.2
*Myotis auriculus*	0.0	0.0	0.0
*Idionycteris phyllotis*	0.0	0.0	0.0
*Myotis occultus*	0.0	0.0	0.0
*Pipistrellus hesperus*	0.0	0.0	0.0

**Table 2:** The expected conservation status of the North American bats susceptible to White-Nose syndrome spread according to the population-reduction scenarios of Table 1 and the IUCN^a^ criteria.

			Scenarios	
Species	Current status	Pessimistic	Intermediate	Optimistic
*Myotis leibii*	LC	CE	CE	LC
*Myotis sodalis* b	EN	CE	CE	EN
*Myotis grisescens*	NT	CE	EN	LC
*Corynorhinus rafinesquii*	LC	CE	EN	LC
*Myotis austroriparius*	LC	CE	EN	LC
*Nycticeius humeralis*	LC	EN	VU	LC
*Pipistrellus subflavus*	LC	EN	VU	LC
*Myotis septentrionalis*	LC	VU	VU	LC
*Eptesicus fuscus*	LC	LC	LC	LC
*Myotis lucifugus*	LC	LC	LC	LC
*Myotis vellifer*	LC	LC	LC	LC
*Corynorhinus townsendii*	LC	LC	LC	LC
*Myotis evotis*	LC	LC	LC	LC
*Euderma maculatum*	LC	LC	LC	LC
*Myotis volans*	LC	LC	LC	LC
*Myotis californicus*	LC	LC	LC	LC
*Myotis yumanensis*	LC	LC	LC	LC
*Myotis ciliolabrum*	LC	LC	LC	LC
*Myotis keenii*	LC	LC	LC	LC
*Antrozous pallidus*	LC	LC	LC	LC
*Myotis thysanodes*	LC	LC	LC	LC
*Myotis auriculus*	LC	LC	LC	LC
*Idionycteris phyllotis*	LC	LC	LC	LC
*Myotis occultus*	LC	LC	LC	LC
*Pipistrellus hesperus*	LC	LC	LC	LC

a International Union for Conservation of Nature and Natural Resources.

b The expected conservation status of *Myotis sodalis* is not solely based on the estimated population decline caused by the potential spread of White-Nose syndrome. We also used an estimated population decline of 50% calculated before 2008 by the IUCN, the main cause of which was human disturbance in caves. This represents an overall population decline of 88.15, 78.15, and 61.55 for the Pessimistic, Intermediate and Optimistic scenarios, respectively.

## Discussion

The predicted expansion of WNS in North America is congruent with its currently known distribution (Figure 1, see figure 2 in [35]). However, the predicted spread revealed important new areas for invasion, such as the Mississippi basin and the southwestern part of the Appalachian Mountains. With a range of 9–11 species per cell (Figure S2), these regions exhibit a large number of bat species in relation to other Nearctic regions.

This potential spread is similar to that found in other modeling exercises [35], [41], despite of some key differences. Flory et al. [35] suggest a smaller geographic distribution, which would have been expected since their main objective was not to detect the potential WNS distribution but its actual distribution. As our purpose was to predict the potential spread of WNS, we used point localities from the native fungus range (based on the novel pathogenesis hypothesis [11]) to calibrate the model, and the invaded fungus range to validate the model, attempting to produce a more accurate potential spread for the invaded region. In other modeling exercises, researchers have used a diffusive model that considers climatic and geographic features; the spread of WNS towards the western of United States of America (USA) was greater than predicted in our model [41]. Once again, this discrepancy between spread scenarios can be assigned to the purpose of the study [41], which was to understand the spread pattern reflected in the selection of sample units (USA counties) and the extent of the study (only USA). As far as we know, our study is the first to infer the direct effects of a spread in WNS upon North American bats.

Before the onset of the WNS, the mammalian groups most affected by infectious diseases were those with higher proportions of domesticated species (i.e., Artiodactyla and Carnivora; [9]). Unfortunately, our results suggest that this picture will probably change for North American bats. Our Pessimistic scenario indicated that eight species would experience an impact strong enough to categorize them as threatened. For example, *M. leibii* will probably be the most affected one, with more than 96% of population decline, based on the Pessimistic scenario and would be considered as CE. The current distribution of this species is very congruent with the potential spread of WNS, encompassing all the Appalachian Mountains until the Arkansas River. The populations of *M. leibii* were considered stable before the onset of WNS, but some populations are already experiencing negative population trends [17], [42]. Secondly, *M. sodalis*, with over 88% of estimated population decline and classified as CE. According to our most pessimistic scenario, this species would experience a population reduction around 76%, however this species had already lost 50% of its original population before the outbreak for WNS, mainly due to human disturbance at winter caves [17]. Accessibility restrictions have apparently stabilized its total population since 1983 [17], [22], but unfortunately, recent studies have shown that populations of *M. sodalis* are experiencing negative effects because of the disease, with local and regional extirpation being expected in a short term, threatening years of conservation efforts [22], [43].

*M. grisescens* appeared in third place with 83% of population decline and would be classified as CE. Its geographic distribution encompasses the southernmost Appalachian Mountains including part of the Mississippi and Ohio Rivers, reaching the Gulf of Mexico. Until the 1980s, population declines for *M. grisescens* exceeded 50%, however, its population was increasing before the onset of WNS due to successful conservation programs [17]. The fourth most affected species was *C. rafinesquii* with 80% of population decline and would be classified as CE. This species occurs mostly in southern USA and its current population was considered increasing, but there were missing data on many parts of its distribution, even before the outbreak of WNS [17]. The remaining species classified in a threat category (*M. austroriparius, M. septentrionalis, Nycticeius humeralis,* and *Pipistrellus subflavus)* presented stable populations before the onset of WNS [17], but *M. septentrionalis* and *P. subflavus* are confirmed with negative population trends due to WNS [42]. We discussed the results of WNS effects on species conservation status based only on the Pessimistic scenario, since this is probably the best way to board all potential scenarios (precautionary principle).

Since the outbreak of WNS in 2006, the scientific community has directed efforts towards identifying the etiological agent [44], uncovering the disease origin [11], [45], understanding the mortality mechanism [11], [46], and the factors most associated with the disease distribution [35], [42], [47]. Recently, much effort has been directed towards understanding the disease impact on host populations [19], [20], [21], [22], [43]. For example, some studies used modeling approaches to predict these impacts [19], [43], however, none of these have directly assessed the potential consequences of WNS to the conservation status of all susceptible bats, as we have done here. This is particularly worrying for a pathogen such as *P. destructans* with such a rapid geographic expansion and high pathogenicity.

Several local factors are associated with WNS [42], [48]. Local processes, such as distance between caves and colony size are important to establish the timing of host mortality by WNS [48]. The most important factors for disease persistence are host sociality, colony size and microclimate within the hibernaculum [42]. WNS effects on one species (*M. lucifugus*) could also change the spatial and temporal structure of local bats communities by competitive release [49]. However, these factors are more important to determine the local geographic distribution of the fungus, while the expansion of the disease toward North America depends more on factors at broader spatial scales, such as land classes, climate, and topography [35], [41]. Thus, since the purpose of our study was to investigate the fungus spread and its consequence to the conservation of all North American hibernating bats, we did not include the effect of local processes in our analysis.

Niche theory is broadly used to predict potential areas for biological invasions and disease outbreaks [24], [25], [50]. However, the use of niche theory to estimate diseases spread and subsequently assess the possible disease impact on the conservation status of certain species represents a very recent development. We suggest that the integration of different scales – i.e., local to regional encompassing one to several species – is essential to mitigate the disease effects. However, further advances are required considering a broad-scale approach such as ours: integrating population data, understanding whether there is a phylogenetic signal in host susceptibility, interaction effects of the disease expansion with climate change, and whether hosts could recover from the disease (see [51]).

Finally, our analysis suggests that WNS will spread to the Appalachian Mountains, coastal areas of northeastern North America, the Mississippi basin, and the Rocky Mountains. Therefore, both hibernating and summer sites for bat species within those areas should be particularly managed, including research efforts to control human accessibility and understand population parameters, such as mortality, survival, and dispersal. Additionally, the eight species identified as threatened under the Pessimistic scenario should receive special attention in conservation plans.

## Supporting Information

Figure S1 **Diagram illustrating the method used to evaluate the population decline of a given bat species based on the impact of White-nose Syndrome.** We subtracted the geographic distribution of the hypothetical species “A” by the disease spread. There were three types of population reduction: i) Pessimistic, each cell for the disease corresponded to an impact of 0.99 on the species local population; ii) Intermediate, in which the impact was of 0.73; and iii) Optimistic, with an impact of 0.3. All these values corresponded to maximum, mean and minimum values of mortality rates for *M. lucifugus* due to WNS collected in hibernating sites (see methods section for more details).
(TIF)

Figure S2 **The geographic distribution of hibernating bat species richness in North America.**
(TIF)

Table S1 **Geographic coordinates for sites with the fungus Pseudogymnoascus destructans.** For North American sites, we used the central points of the counties where the disease has been confirmed (compiled from [18]; March 2014). European sites were compiled from [30].
(DOCX)
