# Supplementary figures and images for: The Potential Impact of White-Nose Syndrome on the Conservation Status of North American Bats
Source: PLoS One. 2014 Sep 9;9(9):e107395. doi: 10.1371/journal.pone.0107395 (PMC4159351; doi:10.1371/journal.pone.0107395)

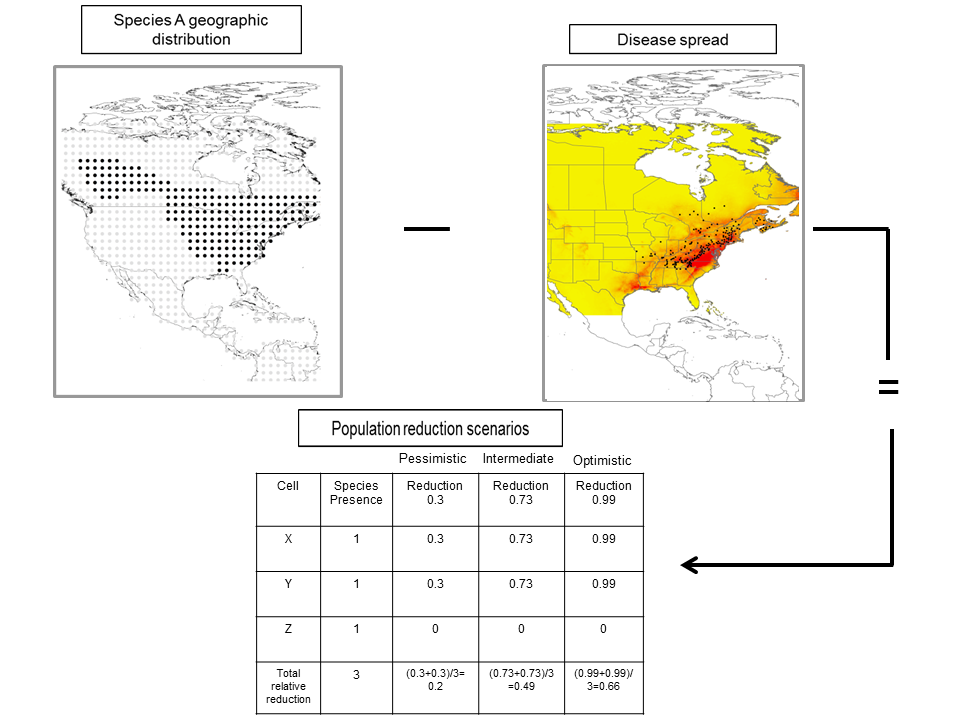

Supplement: Figure S1 — Diagram illustrating the method used to evaluate the population decline of a given bat species based on the impact of White-nose Syndrome. We subtracted the geographic distribution of the hypothetical species “A” by the disease spread. There were three types of population reduction: i) Pessimistic, each cell for the disease corresponded to an impact of 0.99 on the species local population; ii) Intermediate, in which the impact was of 0.73; and iii) Optimistic, with an impact of 0.3. All these values corresponded to maximum, mean and minimum values of mortality rates for M. lucifugus due to WNS collected in hibernating sites (see methods section for more details). (TIF) [file pone.0107395.s001.tif]

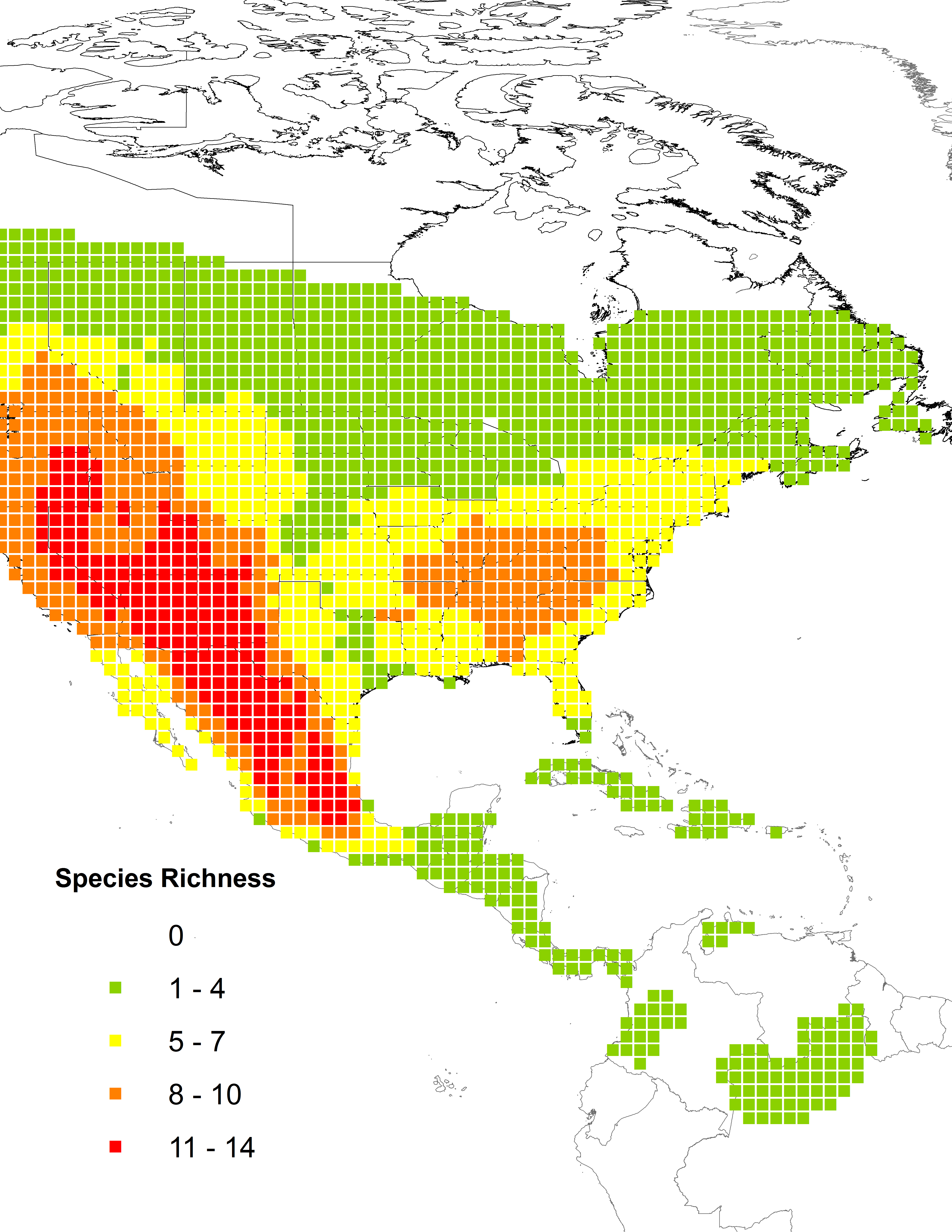

Supplement: Figure S2 — The geographic distribution of hibernating bat species richness in North America. (TIF) [file pone.0107395.s002.tif]
