# Supplementary material for: The Potential Impact of White-Nose Syndrome on the Conservation Status of North American Bats
Source: PLoS One. 2014 Sep 9;9(9):e107395. doi: 10.1371/journal.pone.0107395 (PMC4159351; doi:10.1371/journal.pone.0107395)
Supplement: Table S1 — Geographic coordinates for sites with the fungus Pseudogymnoascus destructans. For North American sites, we used the central points of the counties where the disease has been confirmed (compiled from [18]; March 2014). European sites were compiled from [30]. (DOCX) [file pone.0107395.s003.docx]

| Region | Latitude | Longitude |
| --- | --- | --- |
| Native | 41.9 | 27.9 |
| Native | 42.6 | 2.2 |
| Native | 44.8 | 1.6 |
| Native | 45 | 2 |
| Native | 45.4 | 25.2 |
| Native | 46.2 | 18.1 |
| Native | 46.8 | 16 |
| Native | 46.8 | 22.6 |
| Native | 47 | 22.4 |
| Native | 47.1 | 17.6 |
| Native | 47.2 | 1.4 |
| Native | 47.2 | 5.7 |
| Native | 47.3 | 6.2 |
| Native | 47.7 | -2.1 |
| Native | 47.9 | 6.8 |
| Native | 48.3 | 7.1 |
| Native | 48.3 | 5.7 |
| Native | 48.5 | 20.5 |
| Native | 48.5 | 6.9 |
| Native | 48.7 | 26.6 |
| Native | 48.8 | 26.6 |
| Native | 48.9 | 0.3 |
| Native | 49.1 | 4.1 |
| Native | 49.1 | 6.6 |
| Native | 49.5 | 5.2 |
| Native | 49.7 | 7.4 |
| Native | 49.8 | 5.3 |
| Native | 49.8 | 9.6 |
| Native | 49.9 | 7.4 |
| Native | 50.3 | 5.9 |
| Native | 50.4 | 3.5 |
| Native | 50.6 | 2.5 |
| Native | 50.7 | 13.7 |
| Native | 50.8 | 16.7 |
| Native | 50.8 | 5.6 |
| Native | 50.8 | 5.7 |
| Native | 50.9 | 7.5 |
| Native | 50.9 | 13.3 |
| Native | 51.2 | 8.1 |
| Native | 51.6 | 10.5 |
| Native | 51.7 | 10.3 |
| Native | 51.8 | 10.8 |
| Native | 52 | 5.8 |
| Native | 52 | 5.7 |
| Native | 52.1 | 4.3 |
| Native | 52.1 | 8.2 |
| Native | 52.2 | 8 |
| Native | 52.3 | 9.5 |
| Native | 52.3 | 9.4 |
| Native | 56.4 | 9.1 |
| Native | 59.3 | 24.6 |
| Invaded | 37 | -84.5 |
| Invaded | 37 | -86.5 |
| Invaded | 39 | -94.4 |
| Invaded | 44 | -65.5 |
| Invaded | 50 | -74.3 |
| Invaded | 34.7 | -83.1 |
| Invaded | 34.7 | -84.4 |
| Invaded | 34.7 | -85.2 |
| Invaded | 35.1 | -85.6 |
| Invaded | 35.2 | -82.7 |
| Invaded | 35.2 | -87.4 |
| Invaded | 35.3 | -82.7 |
| Invaded | 35.4 | -81.9 |
| Invaded | 35.4 | -85.7 |
| Invaded | 35.6 | -82.0 |
| Invaded | 35.6 | -82.5 |
| Invaded | 35.6 | -87.9 |
| Invaded | 35.7 | -85.4 |
| Invaded | 35.8 | -86.1 |
| Invaded | 35.8 | -87.5 |
| Invaded | 35.9 | -82.3 |
| Invaded | 35.9 | -85.4 |
| Invaded | 35.9 | -85.8 |
| Invaded | 36.0 | -81.9 |
| Invaded | 36.1 | -87.4 |
| Invaded | 36.2 | -82.8 |
| Invaded | 36.2 | -82.4 |
| Invaded | 36.2 | -85.5 |
| Invaded | 36.2 | -92.7 |
| Invaded | 36.3 | -82.1 |
| Invaded | 36.3 | -83.8 |
| Invaded | 36.3 | -85.3 |
| Invaded | 36.4 | -83.3 |
| Invaded | 36.4 | -84.1 |
| Invaded | 36.4 | -84.9 |
| Invaded | 36.4 | -85.6 |
| Invaded | 36.5 | -87.4 |
| Invaded | 36.5 | -82.3 |
| Invaded | 36.7 | -83 |
| Invaded | 36.8 | -87.9 |
| Invaded | 36.8 | -81.5 |
| Invaded | 36.8 | -82 |
| Invaded | 36.8 | -83.3 |
| Invaded | 36.8 | -84.1 |
| Invaded | 36.9 | -87.5 |
| Invaded | 37.0 | -82.7 |
| Invaded | 37.1 | -81.6 |
| Invaded | 37.1 | -81.1 |
| Invaded | 37.1 | -82.8 |
| Invaded | 37.2 | -86.2 |
| Invaded | 37.3 | -80.7 |
| Invaded | 37.3 | -85.9 |
| Invaded | 37.4 | -81.1 |
| Invaded | 37.4 | -84.3 |
| Invaded | 37.4 | -88.6 |
| Invaded | 37.5 | -80.2 |
| Invaded | 37.5 | -88.6 |
| Invaded | 37.5 | -88.3 |
| Invaded | 37.6 | -80.6 |
| Invaded | 37.6 | -85.5 |
| Invaded | 37.6 | -93 |
| Invaded | 37.9 | -80.5 |
| Invaded | 37.9 | -90.9 |
| Invaded | 37.9 | -91.3 |
| Invaded | 38.0 | -81.1 |
| Invaded | 38.0 | -79.8 |
| Invaded | 38.2 | -80.1 |
| Invaded | 38.3 | -86.6 |
| Invaded | 38.3 | -79.6 |
| Invaded | 38.3 | -83.1 |
| Invaded | 38.3 | -90.2 |
| Invaded | 38.4 | -91.1 |
| Invaded | 38.5 | -79.0 |
| Invaded | 38.6 | -82.5 |
| Invaded | 38.6 | -86.1 |
| Invaded | 38.7 | -79.4 |
| Invaded | 38.8 | -79.9 |
| Invaded | 39.0 | -78.9 |
| Invaded | 39.1 | -79.6 |
| Invaded | 39.1 | -79.3 |
| Invaded | 39.2 | -86.6 |
| Invaded | 39.3 | -77.9 |
| Invaded | 39.3 | -82 |
| Invaded | 39.4 | -84.2 |
| Invaded | 39.5 | -79.4 |
| Invaded | 39.6 | -78.7 |
| Invaded | 39.6 | -77.8 |
| Invaded | 39.9 | -79.7 |
| Invaded | 39.9 | -78.2 |
| Invaded | 39.9 | -83.4 |
| Invaded | 40.0 | -79.1 |
| Invaded | 40.3 | -79.6 |
| Invaded | 40.3 | -75.2 |
| Invaded | 40.3 | -83.3 |
| Invaded | 40.4 | -78.0 |
| Invaded | 40.5 | -78.4 |
| Invaded | 40.6 | -77.6 |
| Invaded | 40.8 | -76.7 |
| Invaded | 40.8 | -75.0 |
| Invaded | 40.8 | -74.6 |
| Invaded | 40.8 | -82.2 |
| Invaded | 40.9 | -77.9 |
| Invaded | 40.9 | -75.8 |
| Invaded | 40.9 | -81.9 |
| Invaded | 41.0 | -80.4 |
| Invaded | 41.0 | -75.4 |
| Invaded | 41.1 | -76.1 |
| Invaded | 41.2 | -73.4 |
| Invaded | 41.2 | -81.8 |
| Invaded | 41.3 | -77.1 |
| Invaded | 41.3 | -74.3 |
| Invaded | 41.3 | -73.8 |
| Invaded | 41.3 | -88.9 |
| Invaded | 41.4 | -75.7 |
| Invaded | 41.4 | -72.1 |
| Invaded | 41.4 | -83.2 |
| Invaded | 41.7 | -77.3 |
| Invaded | 41.7 | -74.8 |
| Invaded | 41.8 | -72.8 |
| Invaded | 41.8 | -73.3 |
| Invaded | 41.8 | -74.3 |
| Invaded | 42.1 | -73.7 |
| Invaded | 42.1 | -72.7 |
| Invaded | 42.2 | -73.3 |
| Invaded | 42.5 | -72.6 |
| Invaded | 42.5 | -74.0 |
| Invaded | 42.5 | -74.5 |
| Invaded | 42.6 | -77.8 |
| Invaded | 42.7 | -78.8 |
| Invaded | 42.8 | -74.5 |
| Invaded | 42.9 | -73.2 |
| Invaded | 43.0 | -76.2 |
| Invaded | 43.0 | -72.7 |
| Invaded | 43.2 | -71.7 |
| Invaded | 43.3 | -73.5 |
| Invaded | 43.5 | -79.9 |
| Invaded | 43.5 | -74.0 |
| Invaded | 43.5 | -73.1 |
| Invaded | 43.5 | -72.6 |
| Invaded | 43.6 | -74.5 |
| Invaded | 43.7 | -79.8 |
| Invaded | 43.9 | -72.4 |
| Invaded | 43.9 | -71.9 |
| Invaded | 44.0 | -80.2 |
| Invaded | 44.0 | -75.9 |
| Invaded | 44.0 | -73.8 |
| Invaded | 44.2 | -72.7 |
| Invaded | 44.3 | -81.3 |
| Invaded | 44.3 | -64.9 |
| Invaded | 44.4 | -80.8 |
| Invaded | 44.4 | -70.8 |
| Invaded | 44.4 | -65.9 |
| Invaded | 44.4 | -64.7 |
| Invaded | 44.5 | -79.7 |
| Invaded | 44.6 | -78.2 |
| Invaded | 44.6 | -74.3 |
| Invaded | 44.6 | -72.6 |
| Invaded | 44.6 | -71.3 |
| Invaded | 44.7 | -73.7 |
| Invaded | 44.7 | -65.3 |
| Invaded | 44.8 | -77.7 |
| Invaded | 44.8 | -63.2 |
| Invaded | 44.8 | -64.7 |
| Invaded | 45.0 | -63.9 |
| Invaded | 45.2 | -72.2 |
| Invaded | 45.5 | -77.2 |
| Invaded | 45.5 | -71.0 |
| Invaded | 45.5 | -65.3 |
| Invaded | 45.6 | -75.9 |
| Invaded | 45.8 | -67.1 |
| Invaded | 46.2 | -71.7 |
| Invaded | 46.3 | -63.5 |
| Invaded | 46.4 | -66.8 |
| Invaded | 46.5 | -79.1 |
| Invaded | 46.5 | -65.2 |
| Invaded | 46.7 | -66.1 |
| Invaded | 46.8 | -72.5 |
| Invaded | 47.1 | -66.4 |
| Invaded | 47.9 | -80.4 |
| Invaded | 47.9 | -84.5 |
| Invaded | 47.9 | -82.2 |
| Invaded | 48.1 | -77.9 |
| Invaded | 48.7 | -81.3 |
| Invaded | 49.5 | -76.6 |
